# Assessment of Appropriateness of Cardiovascular Preventive Medication in Adults ≥ 75 years

**DOI:** 10.1093/geroni/igaa057.1690

**Published:** 2020-12-16

**Authors:** Milly van der Ploeg, Rosalinde Poortvliet, Jacobijn Gussekloo, Yvonne Drewes

**Affiliations:** 1 Leiden University, Leiden, The Hague, Netherlands; 2 Leiden University Medical Center, Leiden, Zuid-Holland, Netherlands

## Abstract

Physicians are confronted with dilemma’s on cardiovascular preventive medication for older people with complex health problems. With accumulation of diseases, limitations and shortening life expectancy, questions arise for whom cardiovascular preventive treatment is still appropriate (the expected benefits of treatment exceeds the negative consequences by a sufficiently wide margin resulting that treatment is worth doing). There is a need for more guidance in clinical situations. With the RAND/UCLA appropriateness method (RUAM), we investigated the appropriateness of cardiovascular preventive medication in adults ≥ 75 years. The RUAM is a systematic, formalized method, to combine available scientific evidence with the collective judgment of experts. Fourteen interdisciplinary panelists (9 physicians representing 6 medical disciplines, 1 medical ethics expert, 1 pharmacist and 3 older adults [lay man]), discussed and rated the appropriateness of starting and stopping of three medication groups (cholesterol lowering, blood pressure lowering and thrombocyte aggregation inhibitors) for different clinical scenarios (combinations of cardiovascular history, systolic blood pressure, complexity of health problems, age, life-expectancy, side-effects). Depending on the medication group, different patterns of appropriateness judgments across the clinical scenarios were found. In general, absence of cardiovascular disease, presence of complex health problems, a short life-expectancy or hindering side-effects were important factors to judge cardiovascular preventive medication as inappropriate. Results were summarized into colored reading maps. These findings can offer more guidance in clinical decision making about cardiovascular preventive treatment for adults ≥ 75 years.

